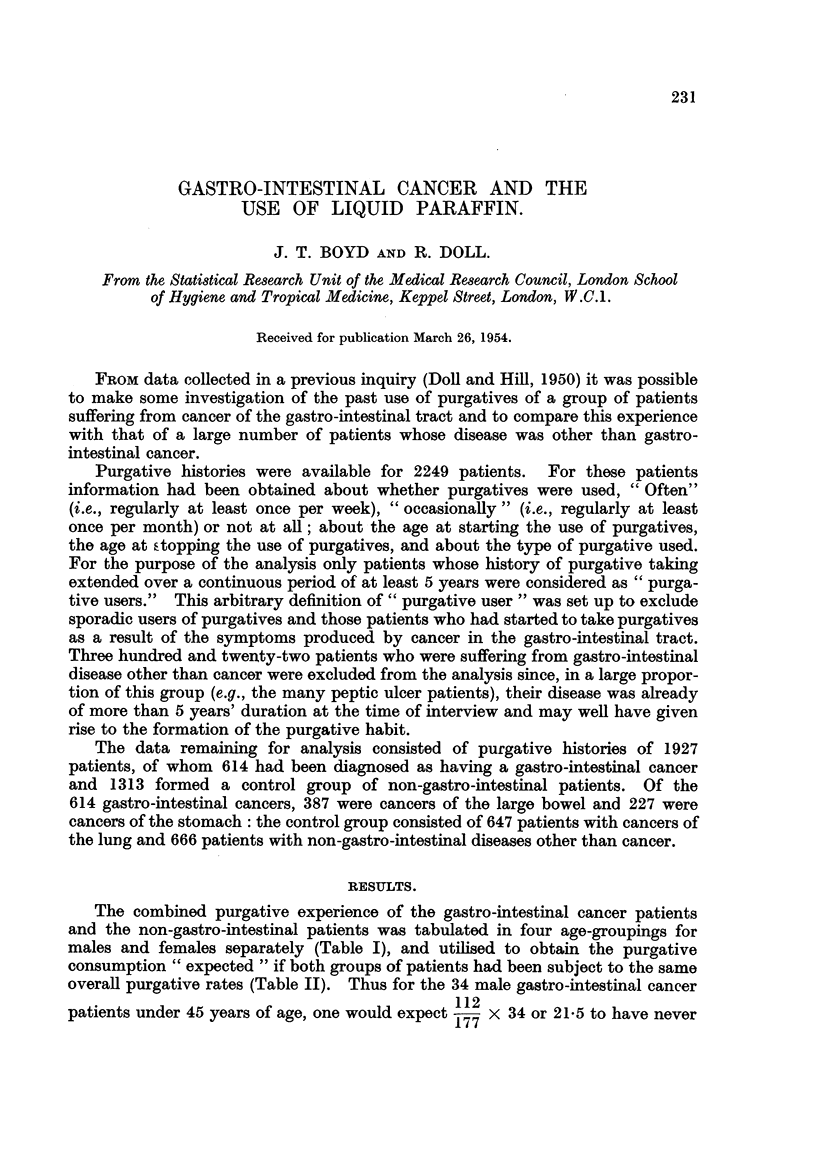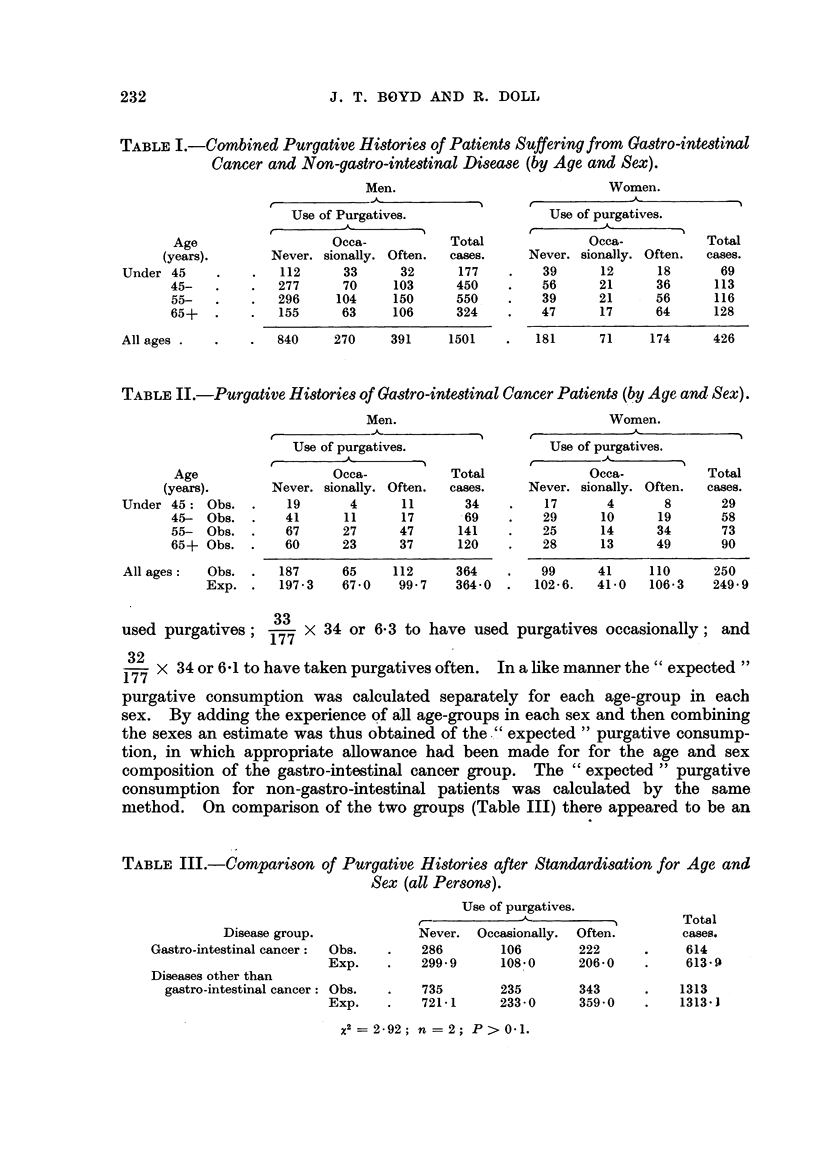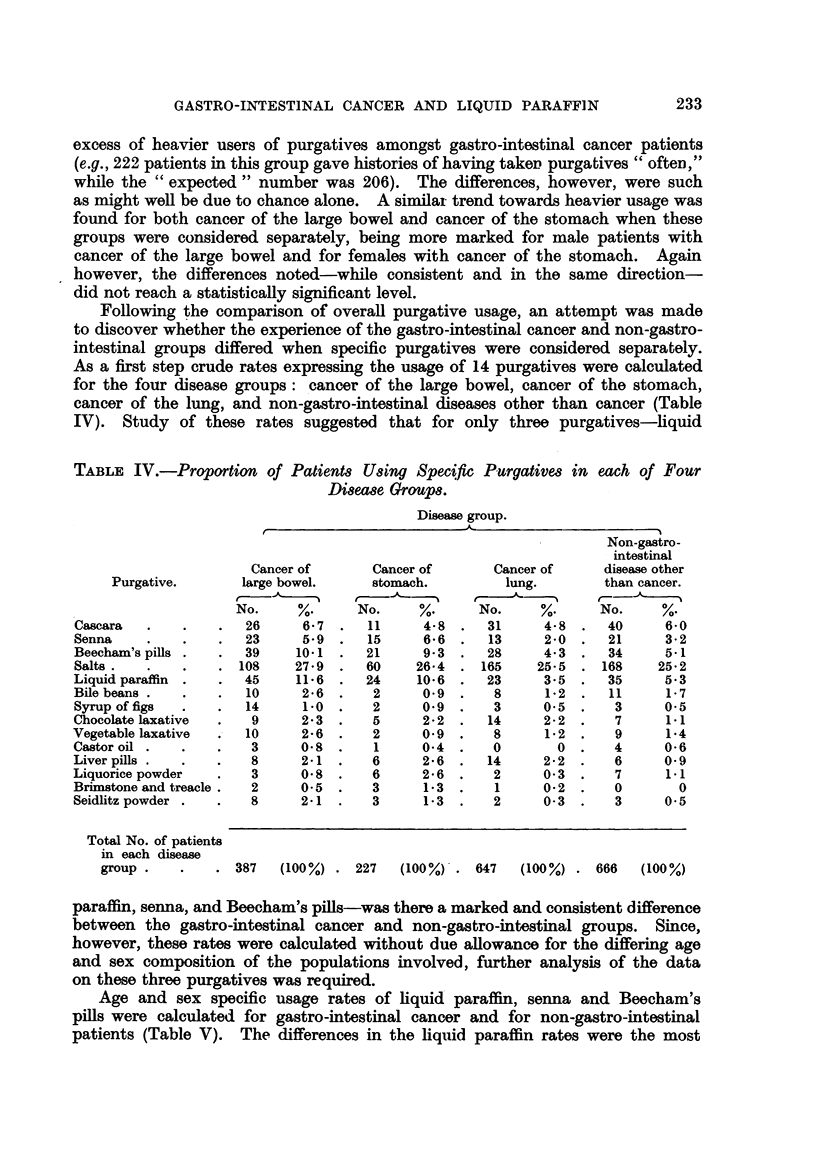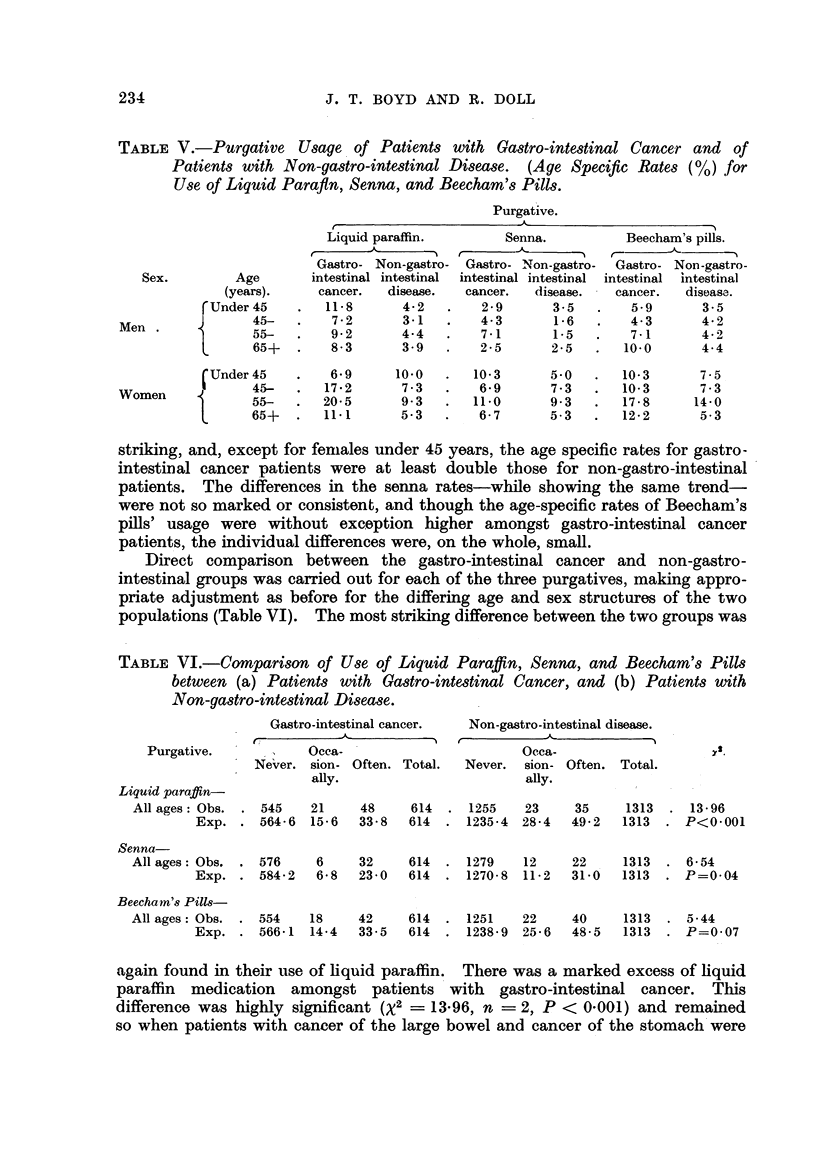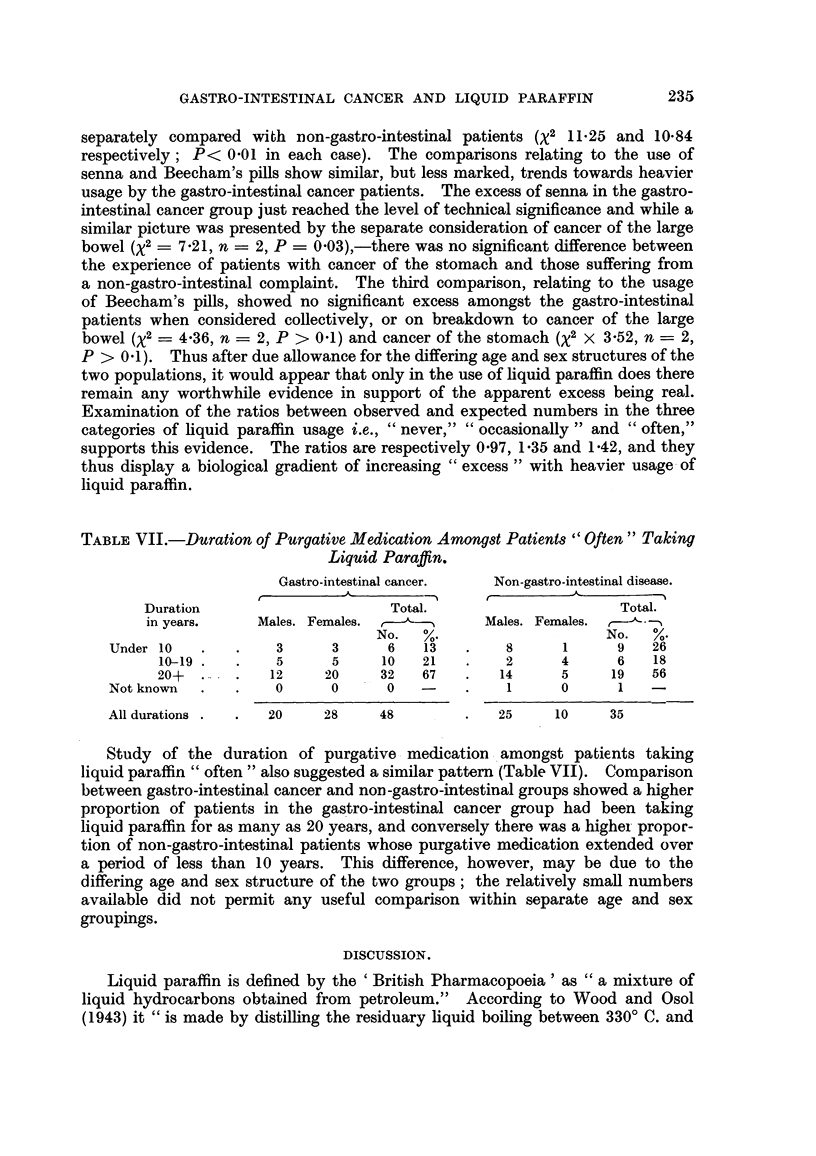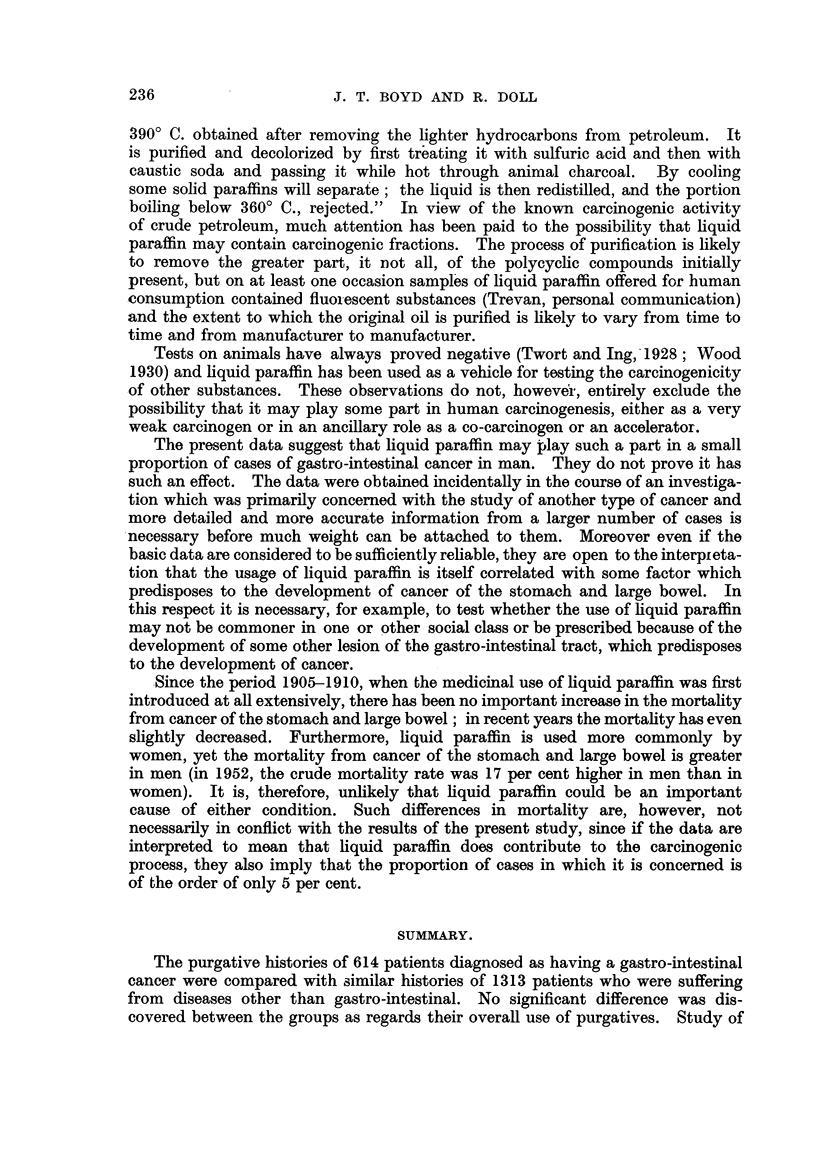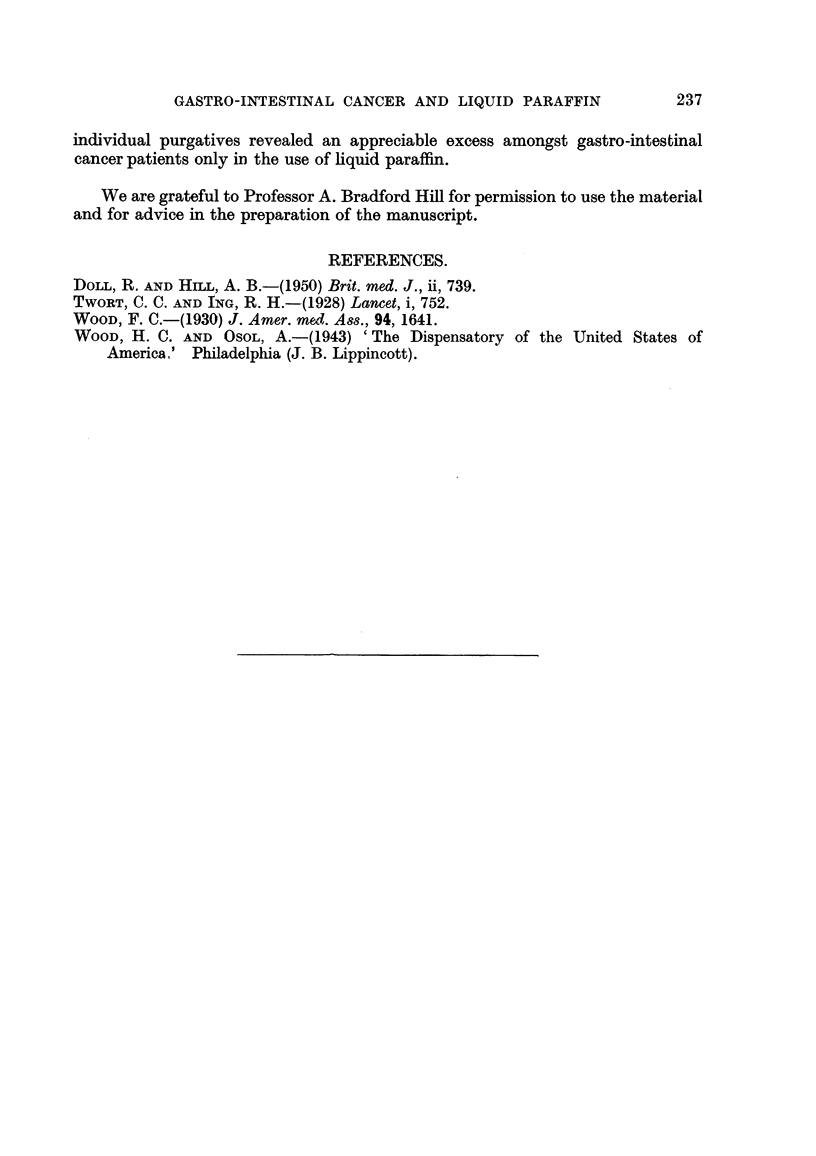# Gastro-Intestinal Cancer and the Use of Liquid Paraffin

**DOI:** 10.1038/bjc.1954.23

**Published:** 1954-06

**Authors:** J. T. Boyd, R. Doll


### GASTRO-INTESTINAL CANCER AND THE USE OF LIQUID PARAFFIN.

J. T. BOYD AND R. DOLL.

*From the Statistical Research Unit of the Medical Research Council, London School of Hygiene and Tropical Medicine, Keppel Street, London, W.C.1.*

Received for publication March 26, 1954.

FROM data collected in a previous inquiry (Doll and Hill, 1950) it was possible to make some investigation of the past use of purgatives of a group of patients suffering from cancer of the gastro-intestinal tract and to compare this experience with that of a large number of patients whose disease was other than gastro-intestinal cancer.

Purgative histories were available for 2249 patients. For these patients information had been obtained about whether purgatives were used, "Often" (*i.e.*, regularly at least once per week), "occasionally" (*i.e.*, regularly at least once per month) or not at all; about the age at starting the use of purgatives, the age at stopping the use of purgatives, and about the type of purgative used. For the purpose of the analysis only patients whose history of purgative taking extended over a continuous period of at least 5 years were considered as "purgative users." This arbitrary definition of "purgative user" was set up to exclude sporadic users of purgatives and those patients who had started to take purgatives as a result of the symptoms produced by cancer in the gastro-intestinal tract. Three hundred and twenty-two patients who were suffering from gastro-intestinal disease other than cancer were excluded from the analysis since, in a large proportion of this group (*e.g.*, the many peptic ulcer patients), their disease was already of more than 5 years' duration at the time of interview and may well have given rise to the formation of the purgative habit.

The data remaining for analysis consisted of purgative histories of 1927 patients, of whom 614 had been diagnosed as having a gastro-intestinal cancer and 1313 formed a control group of non-gastro-intestinal patients. Of the 614 gastro-intestinal cancers, 387 were cancers of the large bowel and 227 were cancers of the stomach : the control group consisted of 647 patients with cancers of the lung and 666 patients with non-gastro-intestinal diseases other than cancer.

### RESULTS.

The combined purgative experience of the gastro-intestinal cancer patients and the non-gastro-intestinal patients was tabulated in four age-groupings for males and females separately (Table I), and utilised to obtain the purgative consumption "expected" if both groups of patients had been subject to the same overall purgative rates (Table II). Thus for the 34 male gastro-intestinal cancer patients under 45 years of age, one would expect $\frac{112}{177} \times 34$ or 21·5 to have never

TABLE I.—*Combined Purgative Histories of Patients Suffering from Gastro-intestinal Cancer and Non-gastro-intestinal Disease (by Age and Sex).*

| Age (years). | Men. Use of Purgatives. Never. | Occasionally. | Often. | Total cases. | Women. Use of purgatives. Never. | Occasionally. | Often. | Total cases. |
|---|---|---|---|---|---|---|---|---|
| Under 45 | 112 | 33 | 32 | 177 | 39 | 12 | 18 | 69 |
| 45– | 277 | 70 | 103 | 450 | 56 | 21 | 36 | 113 |
| 55– | 296 | 104 | 150 | 550 | 39 | 21 | 56 | 116 |
| 65+ | 155 | 63 | 106 | 324 | 47 | 17 | 64 | 128 |
| All ages | 840 | 270 | 391 | 1501 | 181 | 71 | 174 | 426 |

TABLE II.—*Purgative Histories of Gastro-intestinal Cancer Patients (by Age and Sex).*

| Age (years). | | Men. Use of purgatives. Never. | Occasionally. | Often. | Total cases. | Women. Use of purgatives. Never. | Occasionally. | Often. | Total cases. |
|---|---|---|---|---|---|---|---|---|---|
| Under 45 : | Obs. | 19 | 4 | 11 | 34 | 17 | 4 | 8 | 29 |
| 45– | Obs. | 41 | 11 | 17 | 69 | 29 | 10 | 19 | 58 |
| 55– | Obs. | 67 | 27 | 47 | 141 | 25 | 14 | 34 | 73 |
| 65+ | Obs. | 60 | 23 | 37 | 120 | 28 | 13 | 49 | 90 |
| All ages : | Obs. | 187 | 65 | 112 | 364 | 99 | 41 | 110 | 250 |
| | Exp. | 197·3 | 67·0 | 99·7 | 364·0 | 102·6. | 41·0 | 106·3 | 249·9 |

used purgatives ; $\frac{33}{177} \times 34$ or 6·3 to have used purgatives occasionally ; and $\frac{32}{177} \times 34$ or 6·1 to have taken purgatives often. In a like manner the " expected " purgative consumption was calculated separately for each age-group in each sex. By adding the experience of all age-groups in each sex and then combining the sexes an estimate was thus obtained of the " expected " purgative consumption, in which appropriate allowance had been made for for the age and sex composition of the gastro-intestinal cancer group. The " expected " purgative consumption for non-gastro-intestinal patients was calculated by the same method. On comparison of the two groups (Table III) there appeared to be an

TABLE III.—*Comparison of Purgative Histories after Standardisation for Age and Sex (all Persons).*

| Disease group. | | Use of purgatives. Never. | Occasionally. | Often. | Total cases. |
|---|---|---|---|---|---|
| Gastro-intestinal cancer : | Obs. | 286 | 106 | 222 | 614 |
| | Exp. | 299·9 | 108·0 | 206·0 | 613·9 |
| Diseases other than gastro-intestinal cancer : | Obs. | 735 | 235 | 343 | 1313 |
| | Exp. | 721·1 | 233·0 | 359·0 | 1313·1 |

$\chi^2 = 2\cdot92$ ; $n = 2$ ; $P > 0\cdot1$.

excess of heavier users of purgatives amongst gastro-intestinal cancer patients (*e.g.*, 222 patients in this group gave histories of having taken purgatives "often," while the "expected" number was 206). The differences, however, were such as might well be due to chance alone. A similar trend towards heavier usage was found for both cancer of the large bowel and cancer of the stomach when these groups were considered separately, being more marked for male patients with cancer of the large bowel and for females with cancer of the stomach. Again however, the differences noted—while consistent and in the same direction—did not reach a statistically significant level.

Following the comparison of overall purgative usage, an attempt was made to discover whether the experience of the gastro-intestinal cancer and non-gastro-intestinal groups differed when specific purgatives were considered separately. As a first step crude rates expressing the usage of 14 purgatives were calculated for the four disease groups: cancer of the large bowel, cancer of the stomach, cancer of the lung, and non-gastro-intestinal diseases other than cancer (Table IV). Study of these rates suggested that for only three purgatives—liquid

TABLE IV.—*Proportion of Patients Using Specific Purgatives in each of Four Disease Groups.*

| Purgative. | Disease group. | | | | | | | |
|---|---|---|---|---|---|---|---|---|
| | Cancer of large bowel. | | Cancer of stomach. | | Cancer of lung. | | Non-gastro-intestinal disease other than cancer. | |
| | No. | %. | No. | %. | No. | %. | No. | %. |
| Cascara | 26 | 6·7 | 11 | 4·8 | 31 | 4·8 | 40 | 6·0 |
| Senna | 23 | 5·9 | 15 | 6·6 | 13 | 2·0 | 21 | 3·2 |
| Beecham's pills | 39 | 10·1 | 21 | 9·3 | 28 | 4·3 | 34 | 5·1 |
| Salts | 108 | 27·9 | 60 | 26·4 | 165 | 25·5 | 168 | 25·2 |
| Liquid paraffin | 45 | 11·6 | 24 | 10·6 | 23 | 3·5 | 35 | 5·3 |
| Bile beans | 10 | 2·6 | 2 | 0·9 | 8 | 1·2 | 11 | 1·7 |
| Syrup of figs | 14 | 1·0 | 2 | 0·9 | 3 | 0·5 | 3 | 0·5 |
| Chocolate laxative | 9 | 2·3 | 5 | 2·2 | 14 | 2·2 | 7 | 1·1 |
| Vegetable laxative | 10 | 2·6 | 2 | 0·9 | 8 | 1·2 | 9 | 1·4 |
| Castor oil | 3 | 0·8 | 1 | 0·4 | 0 | 0 | 4 | 0·6 |
| Liver pills | 8 | 2·1 | 6 | 2·6 | 14 | 2·2 | 6 | 0·9 |
| Liquorice powder | 3 | 0·8 | 6 | 2·6 | 2 | 0·3 | 7 | 1·1 |
| Brimstone and treacle | 2 | 0·5 | 3 | 1·3 | 1 | 0·2 | 0 | 0 |
| Seidlitz powder | 8 | 2·1 | 3 | 1·3 | 2 | 0·3 | 3 | 0·5 |
| Total No. of patients in each disease group | 387 | (100%) | 227 | (100%) | 647 | (100%) | 666 | (100%) |

paraffin, senna, and Beecham's pills—was there a marked and consistent difference between the gastro-intestinal cancer and non-gastro-intestinal groups. Since, however, these rates were calculated without due allowance for the differing age and sex composition of the populations involved, further analysis of the data on these three purgatives was required.

Age and sex specific usage rates of liquid paraffin, senna and Beecham's pills were calculated for gastro-intestinal cancer and for non-gastro-intestinal patients (Table V). The differences in the liquid paraffin rates were the most

TABLE V.—*Purgative Usage of Patients with Gastro-intestinal Cancer and of Patients with Non-gastro-intestinal Disease.* (*Age Specific Rates* (%) *for Use of Liquid Parafln, Senna, and Beecham's Pills.*

| | | Purgative. | | | | | |
|---|---|---|---|---|---|---|---|
| | | Liquid paraffin. | | Senna. | | Beecham's pills. | |
| Sex. | Age (years). | Gastro-intestinal cancer. | Non-gastro-intestinal disease. | Gastro-intestinal cancer. | Non-gastro-intestinal disease. | Gastro-intestinal cancer. | Non-gastro-intestinal disease. |
| Men | Under 45 | 11·8 | 4·2 | 2·9 | 3·5 | 5·9 | 3·5 |
| | 45– | 7·2 | 3·1 | 4·3 | 1·6 | 4·3 | 4·2 |
| | 55– | 9·2 | 4·4 | 7·1 | 1·5 | 7·1 | 4·2 |
| | 65+ | 8·3 | 3·9 | 2·5 | 2·5 | 10·0 | 4·4 |
| Women | Under 45 | 6·9 | 10·0 | 10·3 | 5·0 | 10·3 | 7·5 |
| | 45– | 17·2 | 7·3 | 6·9 | 7·3 | 10·3 | 7·3 |
| | 55– | 20·5 | 9·3 | 11·0 | 9·3 | 17·8 | 14·0 |
| | 65+ | 11·1 | 5·3 | 6·7 | 5·3 | 12·2 | 5·3 |

striking, and, except for females under 45 years, the age specific rates for gastro-intestinal cancer patients were at least double those for non-gastro-intestinal patients. The differences in the senna rates—while showing the same trend—were not so marked or consistent, and though the age-specific rates of Beecham's pills' usage were without exception higher amongst gastro-intestinal cancer patients, the individual differences were, on the whole, small.

Direct comparison between the gastro-intestinal cancer and non-gastro-intestinal groups was carried out for each of the three purgatives, making appropriate adjustment as before for the differing age and sex structures of the two populations (Table VI). The most striking difference between the two groups was

TABLE VI.—*Comparison of Use of Liquid Paraffin, Senna, and Beecham's Pills between* (a) *Patients with Gastro-intestinal Cancer, and* (b) *Patients with Non-gastro-intestinal Disease.*

| Purgative. | | Gastro-intestinal cancer. | | | | Non-gastro-intestinal disease. | | | | $\chi^2$. |
|---|---|---|---|---|---|---|---|---|---|---|
| | | Never. | Occasionally. | Often. | Total. | Never. | Occasionally. | Often. | Total. | |
| *Liquid paraffin*— | | | | | | | | | | |
| All ages : | Obs. | 545 | 21 | 48 | 614 | 1255 | 23 | 35 | 1313 | 13·96 |
| | Exp. | 564·6 | 15·6 | 33·8 | 614 | 1235·4 | 28·4 | 49·2 | 1313 | $P<0·001$ |
| *Senna*— | | | | | | | | | | |
| All ages : | Obs. | 576 | 6 | 32 | 614 | 1279 | 12 | 22 | 1313 | 6·54 |
| | Exp. | 584·2 | 6·8 | 23·0 | 614 | 1270·8 | 11·2 | 31·0 | 1313 | $P=0·04$ |
| *Beecham's Pills*— | | | | | | | | | | |
| All ages : | Obs. | 554 | 18 | 42 | 614 | 1251 | 22 | 40 | 1313 | 5·44 |
| | Exp. | 566·1 | 14·4 | 33·5 | 614 | 1238·9 | 25·6 | 48·5 | 1313 | $P=0·07$ |

again found in their use of liquid paraffin. There was a marked excess of liquid paraffin medication amongst patients with gastro-intestinal cancer. This difference was highly significant ($\chi^2 = 13·96$, $n = 2$, $P < 0·001$) and remained so when patients with cancer of the large bowel and cancer of the stomach were separately compared with non-gastro-intestinal patients ($\chi^2$ 11·25 and 10·84 respectively ; $P < 0{\cdot}01$ in each case). The comparisons relating to the use of senna and Beecham's pills show similar, but less marked, trends towards heavier usage by the gastro-intestinal cancer patients. The excess of senna in the gastro-intestinal cancer group just reached the level of technical significance and while a similar picture was presented by the separate consideration of cancer of the large bowel ($\chi^2 = 7{\cdot}21$, $n = 2$, $P = 0{\cdot}03$),—there was no significant difference between the experience of patients with cancer of the stomach and those suffering from a non-gastro-intestinal complaint. The third comparison, relating to the usage of Beecham's pills, showed no significant excess amongst the gastro-intestinal patients when considered collectively, or on breakdown to cancer of the large bowel ($\chi^2 = 4{\cdot}36$, $n = 2$, $P > 0{\cdot}1$) and cancer of the stomach ($\chi^2 \times 3{\cdot}52$, $n = 2$, $P > 0{\cdot}1$). Thus after due allowance for the differing age and sex structures of the two populations, it would appear that only in the use of liquid paraffin does there remain any worthwhile evidence in support of the apparent excess being real. Examination of the ratios between observed and expected numbers in the three categories of liquid paraffin usage *i.e.*, "never," "occasionally" and "often," supports this evidence. The ratios are respectively 0·97, 1·35 and 1·42, and they thus display a biological gradient of increasing "excess" with heavier usage of liquid paraffin.

TABLE VII.—*Duration of Purgative Medication Amongst Patients "Often" Taking Liquid Paraffin.*

| Duration in years. | Gastro-intestinal cancer. Males. | Females. | Total. No. | %. | Non-gastro-intestinal disease. Males. | Females. | Total. No. | %. |
|---|---|---|---|---|---|---|---|---|
| Under 10 | 3 | 3 | 6 | 13 | 8 | 1 | 9 | 26 |
| 10–19 | 5 | 5 | 10 | 21 | 2 | 4 | 6 | 18 |
| 20+ | 12 | 20 | 32 | 67 | 14 | 5 | 19 | 56 |
| Not known | 0 | 0 | 0 | — | 1 | 0 | 1 | — |
| All durations | 20 | 28 | 48 | | 25 | 10 | 35 | |

Study of the duration of purgative medication amongst patients taking liquid paraffin "often" also suggested a similar pattern (Table VII). Comparison between gastro-intestinal cancer and non-gastro-intestinal groups showed a higher proportion of patients in the gastro-intestinal cancer group had been taking liquid paraffin for as many as 20 years, and conversely there was a higher proportion of non-gastro-intestinal patients whose purgative medication extended over a period of less than 10 years. This difference, however, may be due to the differing age and sex structure of the two groups ; the relatively small numbers available did not permit any useful comparison within separate age and sex groupings.

### DISCUSSION.

Liquid paraffin is defined by the 'British Pharmacopoeia' as "a mixture of liquid hydrocarbons obtained from petroleum." According to Wood and Osol (1943) it "is made by distilling the residuary liquid boiling between 330° C. and

390° C. obtained after removing the lighter hydrocarbons from petroleum. It is purified and decolorized by first treating it with sulfuric acid and then with caustic soda and passing it while hot through animal charcoal. By cooling some solid paraffins will separate ; the liquid is then redistilled, and the portion boiling below 360° C., rejected." In view of the known carcinogenic activity of crude petroleum, much attention has been paid to the possibility that liquid paraffin may contain carcinogenic fractions. The process of purification is likely to remove the greater part, it not all, of the polycyclic compounds initially present, but on at least one occasion samples of liquid paraffin offered for human consumption contained fluorescent substances (Trevan, personal communication) and the extent to which the original oil is purified is likely to vary from time to time and from manufacturer to manufacturer.

Tests on animals have always proved negative (Twort and Ing, 1928 ; Wood 1930) and liquid paraffin has been used as a vehicle for testing the carcinogenicity of other substances. These observations do not, however, entirely exclude the possibility that it may play some part in human carcinogenesis, either as a very weak carcinogen or in an ancillary role as a co-carcinogen or an accelerator.

The present data suggest that liquid paraffin may play such a part in a small proportion of cases of gastro-intestinal cancer in man. They do not prove it has such an effect. The data were obtained incidentally in the course of an investigation which was primarily concerned with the study of another type of cancer and more detailed and more accurate information from a larger number of cases is necessary before much weight can be attached to them. Moreover even if the basic data are considered to be sufficiently reliable, they are open to the interpretation that the usage of liquid paraffin is itself correlated with some factor which predisposes to the development of cancer of the stomach and large bowel. In this respect it is necessary, for example, to test whether the use of liquid paraffin may not be commoner in one or other social class or be prescribed because of the development of some other lesion of the gastro-intestinal tract, which predisposes to the development of cancer.

Since the period 1905–1910, when the medicinal use of liquid paraffin was first introduced at all extensively, there has been no important increase in the mortality from cancer of the stomach and large bowel ; in recent years the mortality has even slightly decreased. Furthermore, liquid paraffin is used more commonly by women, yet the mortality from cancer of the stomach and large bowel is greater in men (in 1952, the crude mortality rate was 17 per cent higher in men than in women). It is, therefore, unlikely that liquid paraffin could be an important cause of either condition. Such differences in mortality are, however, not necessarily in conflict with the results of the present study, since if the data are interpreted to mean that liquid paraffin does contribute to the carcinogenic process, they also imply that the proportion of cases in which it is concerned is of the order of only 5 per cent.

### SUMMARY.

The purgative histories of 614 patients diagnosed as having a gastro-intestinal cancer were compared with similar histories of 1313 patients who were suffering from diseases other than gastro-intestinal. No significant difference was discovered between the groups as regards their overall use of purgatives. Study of individual purgatives revealed an appreciable excess amongst gastro-intestinal cancer patients only in the use of liquid paraffin.

We are grateful to Professor A. Bradford Hill for permission to use the material and for advice in the preparation of the manuscript.

### REFERENCES.

DOLL, R. AND HILL, A. B.—(1950) *Brit. med. J.*, ii, 739.
TWORT, C. C. AND ING, R. H.—(1928) *Lancet*, i, 752.
WOOD, F. C.—(1930) *J. Amer. med. Ass.*, **94**, 1641.
WOOD, H. C. AND OSOL, A.—(1943) 'The Dispensatory of the United States of America.' Philadelphia (J. B. Lippincott).